# Intercalation and structural aspects of macroRAFT agents into MgAl layered double hydroxides

**DOI:** 10.3762/bjnano.7.191

**Published:** 2016-12-15

**Authors:** Dessislava Kostadinova, Ana Cenacchi Pereira, Muriel Lansalot, Franck D’Agosto, Elodie Bourgeat-Lami, Fabrice Leroux, Christine Taviot-Guého, Sylvian Cadars, Vanessa Prevot

**Affiliations:** 1 Université Clermont Auvergne, Université Blaise Pascal, Institut de Chimie de Clermont-Ferrand, BP 10448, F-63000 Clermont-Ferrand, France; 2 CNRS, UMR 6296, ICCF, F-63171 Aubière, France; 3 Univ Lyon, Université Claude Bernard Lyon 1, CPE Lyon, CNRS 5265, Chemistry, Catalysis, Polymers and Processes (C2P2), 43 Bvd du 11 Novembre 1918, 69616 Villeurbanne, France; 4 Institut des Matériaux Jean Rouxel (IMN) - UMR6502, 2 rue de la Houssinière, BP32229 44322 Nantes cedex 3, France

**Keywords:** hybrid materials, hydrophilic copolymers, intercalation, layered double hydroxides, RAFT, solid-state NMR

## Abstract

Increasing attention has been devoted to the design of layered double hydroxide (LDH)-based hybrid materials. In this work, we demonstrate the intercalation by anion exchange process of poly(acrylic acid) (PAA) and three different hydrophilic random copolymers of acrylic acid (AA) and *n*-butyl acrylate (BA) with molar masses ranging from 2000 to 4200 g mol^−1^ synthesized by reversible addition-fragmentation chain transfer (RAFT) polymerization, into LDH containing magnesium(II) and aluminium(III) intralayer cations and nitrates as counterions (MgAl-NO_3_ LDH). At basic pH, the copolymer chains (macroRAFT agents) carry negative charges which allowed the establishment of electrostatic interactions with the LDH interlayer and their intercalation. The resulting hybrid macroRAFT/LDH materials displayed an expanded interlamellar domain compared to pristine MgAl-NO_3_ LDH from 1.36 nm to 2.33 nm. Depending on the nature of the units involved into the macroRAFT copolymer (only AA or AA and BA), the intercalation led to monolayer or bilayer arrangements within the interlayer space. The macroRAFT intercalation and the molecular structure of the hybrid phases were further characterized by Fourier transform infrared (FTIR) and solid-state ^13^C, ^1^H and ^27^Al nuclear magnetic resonance (NMR) spectroscopies to get a better description of the local structure.

## Introduction

Within the emergence of a wide range of organic–inorganic hybrid materials with interesting physical and chemical properties [1], hybrid layered double hydroxides (LDH) have attracted considerable attention in the scientific community [2–3]. LDH matrices are layered materials which can be described by the general formula [M(II)_1−_*_x_*M(III)*_x_*(OH)_2_] [A*^n^*^−^*_x_*_/_*_n_m*H_2_O] that clearly evidences the highly tunable LDH chemical composition depending on the layer cations and the interlayer anions [4]. Owing to their anion exchange capacity, LDH can accommodate in their interlamellar domain a large variety of negatively charged molecular organic species with different functional properties. This opens the way to a wide range of applications such as anion scavengers, adsorbents, heterogeneous catalysts, supports for species of interest in nanomedicine and fillers in polymer matrices [5–7]. For instance, hybrid LDH involving amino acids, peptides, nucleosides, nucleic acids [8–10], biopolymers [11–12] and various drugs [13–15] were investigated to develop efficient systems for therapeutic applications [16–17]. Various dyes (sulfonated spyrospiran, methyl orange, fluorescein pigment red, …) [18–19] were confined by intercalation within LDH for applications as pigments or studies in photophysics or photochromism. Due to their high aspect ratio, their layered feature extended to the nanoscale and their hydroxylated surface, LDH are also particularly interesting for fabricating polymer nanocomposites [20–23]. To favor the dispersion of LDH platelets into polymers, hybrid surfactant (usually dodecyl sulphate)-intercalated LDH were prepared and incorporated into polymer matrices such as polyethylene [24], polypropylene [25], poly(methyl methacrylate) [26], elastomers [27], epoxy polymers [28], poly(ε-caprolactone) [29], polyesters [30], polyurethane [31] and polyimide [21]. Alternatively, Leroux et al. described the preparation of a hybrid LDH phase intercalated by an anionic polymerizable surfactant acting further as an anchor that compatibilizes the inorganic LDH with the polymer matrix during the polymerization [32]. Water soluble macromolecules such as poly(vinyl alcohol), poly(acrylic acid) (PAA), poly(styrene sulfonate) were also intercalated into the layered structure to avoid the use of surfactant molecules which can alter nanocomposite properties [33–34]. To design polymer-intercalated LDH hybrid materials, an elegant alternative consists in performing in situ polymerization of monomer-intercalated LDH. Such approach was applied for instance to make LDH intercalated with vinyl benzene sulfonate [33,35], acrylate anion [10,36–37], sulfopropyl methacrylate [38], or aniline derivative monomers [39]; the polymerization being subsequently induced either by moderate thermal treatment, by initiator addition or thanks to the oxidative LDH layer properties. Localizing the polymerization into the layers of LDH can also be performed by attaching an initiator or a controlling agent and further conduct a free radical polymerization [40–41]. Qu et al. [42] applied the latter strategy by adsorbing 4-(benzodithioyl)-4-cyanopentanoate controlling agent and successfully conducting styrene reversible addition-fragmentation chain transfer (RAFT) polymerization [43] in the LDH matrix. This resulted in an exfoliated LDH/polystyrene nanocomposite with a good control of the molar mass of the polystyrene chains.

Recently, we reported that highly stable dispersions at elevated macroRAFT copolymer concentrations [44] could be obtained by adsorbing statistical copolymers of acrylic acid (AA) and *n*-butyl acrylate (BA) (P(AA-*stat-*BA)) of low molar mass (1800 g mol^−1^) synthesized by RAFT polymerization onto MgAl LDH. In this work, we focus on the possibility to design functional hybrid LDH phases by direct intercalation of similar pre-synthesized macroRAFT agents displaying molar masses in the range of 2000–4200 g mol^−1^. PAA and three copolymers composed of AA and BA units obtained by RAFT were designed to interact with the LDH layers after deprotonation of the AA units at neutral pH. The assembly between these macroRAFT polymers and the LDH layers was investigated using an anionic exchange process and the resulting hybrid materials were thoroughly characterized by a set of solid-state characterization techniques including powder X-ray diffraction (PXRD), transmission and scanning electron microscopy (TEM and SEM, respectively), Fourier transform infrared (FTIR) spectroscopy and solid-state ^13^C, ^1^H and ^27^Al nuclear magnetic resonance (NMR) measurements. A particular attention was paid to the macromolecular anion arrangement into the LDH interlayer domain.

## Results and Discussion

MacroRAFT (co)polymers denoted PAA*_n_*-CTPPA and P(AA*_n_*-*stat*-BA*_n_*)-CTPPA (see Table 1) were synthesized by RAFT (co)polymerization of AA and BA in 1,4-dioxane at 80 °C using 4-cyano-4-thiothiopropylsulfanyl pentanoic acid (CTPPA) as RAFT agent and 4,4'-azobis(4-cyanopentanoic acid) (ACPA) as an initiator.

**Table 1:** Characteristics of the macroRAFT agents synthesized in this work.

macroRAFT	DP_n_^a^	*M*_n_ (g·mol^−1^)	Charge^−^/mol	Abbreviation

PAA-CTPPA	49	3800	50	PAA_49_-CTPPA
P(AA-*stat*-BA)-CTPPA	8.5/8.5	2000	9.5	P(AA_8.5_-*stat*-BA_8.5_)-CTPPA
P(AA-*stat*-BA)-CTPPA	14.5/14.5	3200	15.5	P(AA_14.5_-*stat*-BA_14.5_)-CTPPA
P(AA-*stat*-BA)-CTPPA	19.5/19.5	4200	20.5	P(AA_19.5_-*stat*-BA_19.5_)-CTPPA

^a^Number-average degree of polymerization.

**Figure 1:**
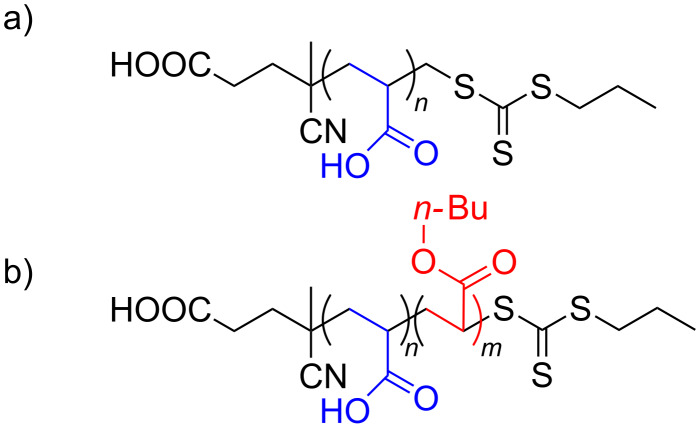
Chemical structures of the macroRAFT agents synthesized in this work: a) PAA*_n_*-CTPPA and b) P(AA*_n_*-*stat*-BA*_n_*)-CTPPA.

These macroRAFT agent compositions were selected with AA units promoting electrostatic interaction with the LDH and BA units to investigate the effect of the insertion of butyl hydrophobic units on the hybrid LDH material structure (Figure 1).

In using AA as hydrophilic monomer, a macroRAFT polymer containing 49 AA units was obtained (*M_n_* = 3800 g mol^−1^; *Ð* = 1.18). During the copolymerization of AA and BA, both monomers were converted at the same rate and were therefore incorporated statistically with negligible composition drift. Well-defined copolymers were obtained with on average 8.5, 14.5 and 20.5 units of each monomer per chain (*M_n_* = 2000; 3200 and 4200 g mol^−1^) and a narrow molar mass distribution (*Ð* = 1.1 ± 0.1). The macroRAFT (co)polymers were neutralized at pH 8 before use, which induced the presence of negative charges on the chains due to the deprotonation of the AA units and of the carboxylic acid end group of CTPPA, leading to 50, 9.5, 15.5 and 19.5 negative charges per mole for PAA_49_-CTPPA, P(AA_8.5_-*stat*-BA_8.5_)-CTPPA, P(AA_14.5_-*stat*-BA_14.5_)-CTPPA and P(AA_19.5_-*stat*-BA_19.5_)-CTPPA, respectively.

The hybrid LDH materials were prepared through anion exchange at room temperature from MgAl-NO_3_ LDH precursor obtained by fast precipitation followed by moderate hydrothermal treatment. Chemical analysis performed on the different samples (Table 2) showed similar Mg/Al ratios, confirming that the layer structure was hardly affected by the anion exchange process.

**Table 2:** Characteristics of the MgAl-NO_3_ LDH precursor and of the hybrid macroRAFT agent-intercalated LDH.

Interlayer anion	d_003_ nm	d_110_ nm	a^a^ nm	c^a^ nm	Mg/Al EDX	ζ mV

NO_3_	0.85	0.152	0.304	2.40	2.8	43
PAA_49_-CTPPA	1.36	0.152	0.304	4.08	2.7	−49
P(AA_8.5_-*stat*-BA_8.5_)-CTPPA	2.326	0.152	0.304	6.90	2.8	−51
P(AA_14.5_-*stat*-BA_14.5_)-CTPPA	2.331	0.152	0.304	6.99	2.8	−53
P(AA_19.5_-*stat*-BA_19.5_)-CTPPA	2.328	0.152	0.304	7.29	2.8	−51

^a^Considering a R-3m space group.

**Figure 2:**
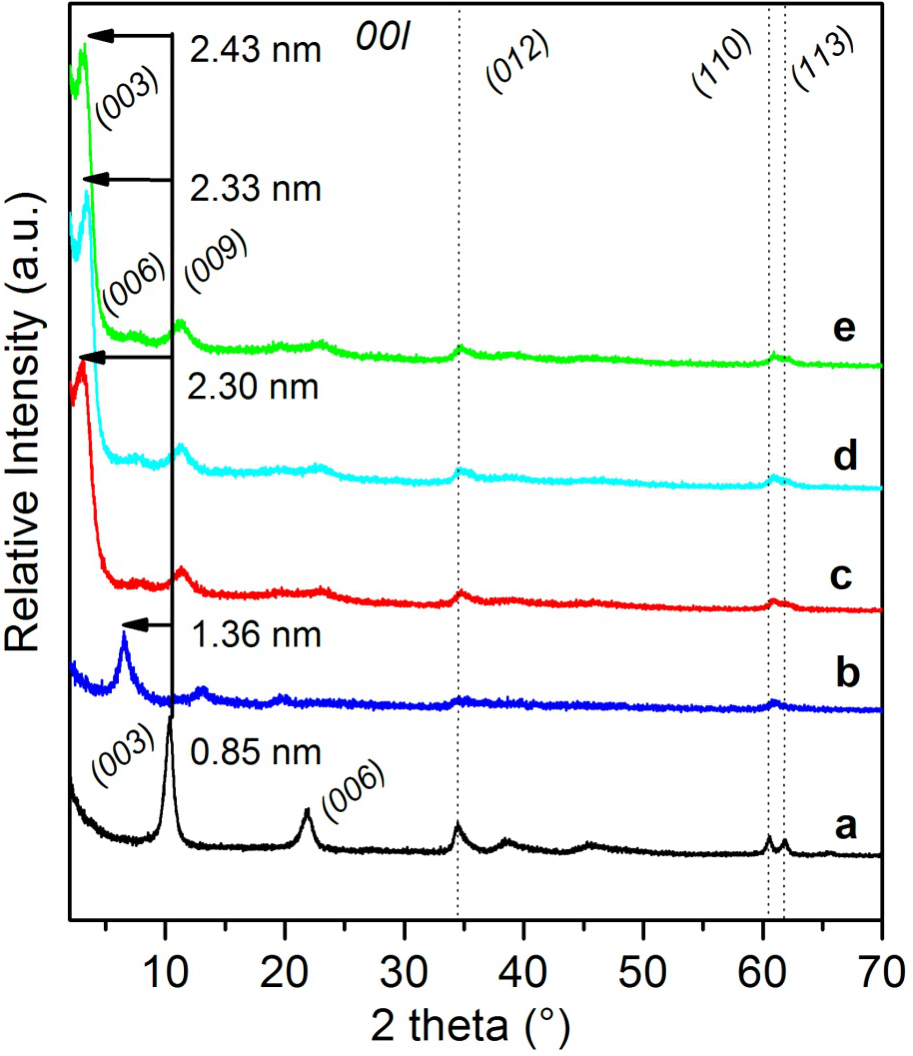
PXRD patterns of: (a) MgAl-NO_3_ LDH and (b–e) macroRAFT agent-intercalated LDH obtained by anion exchange in presence of (b) PAA_49_-CTPPA, (c) P(AA_8.5_-*stat*-BA_8.5_)-CTPPA, (d) P(AA_14.5_-*stat*-BA_14.5_)-CTPPA and (e) P(AA_19.5_-*stat*-BA_19.5_)-CTPPA. The solid line indicates the position of the first 003 reflection in the pristine MgAl-NO_3_ LDH, and the arrows indicate the shift of this reflection due to polymer intercalation.

**Figure 3:**
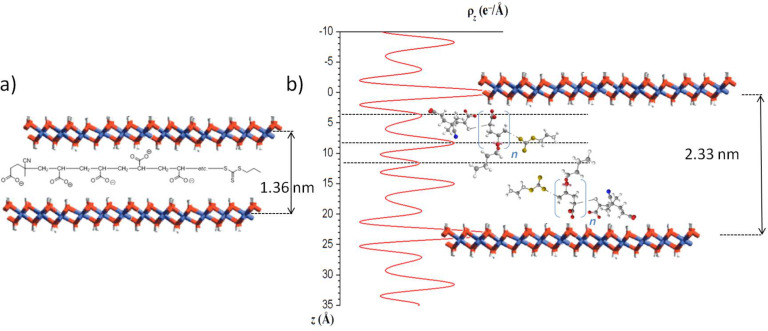
a) Schematic representation of the arrangement of MgAl-PAA_49_-CTPPA, and b) One-dimensional electron density projection along the *c*-stacking axis ρ_(z)_ determined from the analysis of the intensity of 00l X-ray diffraction lines for MgAl-P(AA_8.5_-*stat-*BA_8.5_)-CTPPA and schematic representation of the macromolecule into the LDH interlayer domain.

Compared to the XRD pattern of the LDH precursor (Figure 2) displaying characteristic diffraction lines of a MgAl-NO_3_ phase with a basal spacing of 0.85 nm, a net shift of the 00*l* harmonic reflections to lower values of 2θ below 20° was observed for the LDH-macroRAFT compounds, which is consistent with the replacement of nitrate ions by larger anionic species ensuring the layer surface charge neutralization. For PAA_49_-CTPPA, this modification corresponds to an increase of the interlamellar distance from 0.85 nm to 1.36 nm. This interlamellar distance is similar to that reported in the literature for acrylate anion intercalated LDH and slightly higher than the value obtained for hybrid PAA-intercalated phases formed by in situ free radical polymerization of intercalated AA [37], which can be tentatively attributed to the presence of the CTPPA RAFT end group on the PAA macromolecule. If we consider a layer thickness of 0.21 nm and two hydrogen bond distances between the macromolecule and two adjacent LDH layers (2 × 0.27 nm), the interslab available for the PAA_49_-CTPPA corresponds to 0.6 nm, and indicates an orientation of the macromolecules as a monolayer with the carboxylate groups interacting with the positive adjacent inorganic layers (Figure 3a).

The intercalation of P(AA-*stat*-BA)-CTPPA copolymers led to a larger basal spacing ranging from 2.32 to 2.33 nm, regardless of their molar mass (Table 2). This indicates that the copolymer molar mass did not strongly affect the intercalation and the interlayer arrangement of the macromolecules in between the LDH layers. The crystallinity of the samples was quite low leading to poorly defined X-ray diffraction lines. However, using the Le Bail method (see Supporting Information File 1), we were able to properly reproduce the profile shapes and widths of the reflections and therefore to extract the intensity of the 00*l* diffraction lines reflection, allowing then the calculation of the one-dimensional electron density distribution along the *c*-stacking axis ρ_(z)_. As demonstrated elsewhere, this approach can provide valuable information on the structure of the interlayer space [45]. In the present case, up to 8 isolated 00*l* reflections are expected at low 2θ (<32°) related to the large value of the interlayer distance *d*_00_*_3_* ≈ 2.33 nm and assuming a 3R polytype. Due to the close resemblance of the XRD patterns of P(AA-*stat*-BA)-CTPPA intercalates, the Le Bail analysis led to similar values of the unit cell parameters whatever the molar mass of the copolymer (Table 2), and the extracted intensities of the 00*l* diffraction lines were also found very similar. We therefore assume identical interlayer arrangements such as the one that can be deduced from the 1D plot of MgAl-P(AA_8.5_-*stat*-BA_8.5_)-CTPPA and presented in Figure 3b. The peaks observed match perfectly with a bilayer arrangement of the copolymers. The two most intense peaks are due to the hydroxide layers containing Mg/Al cations. The carboxylate groups from the AA units and the end group of CTPPA would cause maxima at the outer parts of the interlayer space at a distance of ≈0.39 nm from the center of the hydroxide layers which is consistent with hydrogen bond interactions between carboxylate and OH groups: C–O· · ·HO–Mg/Al. The second maximum at a distance of ≈0.44 nm from the latter is attributed to both the carboxylate groups in BA units and the thiopropylsulfanyl groups that would be arranged in an aligned configuration. Finally, the small maximum in the center is attributed to the dangling butyl chains of BA units. This peak assignment is supported by the interatomic distances within the molecules and was determined using the ChemBio 3D Ultra chemical structure drawing program. It is noteworthy that the host LDH layers offer a sufficient surface area per unit charge (0.312 nm^2^/charge) to accommodate the AA subunit (0.06 nm^2^/e) and AA-BA subunit (0.34 nm^2^/e) in the idealistic model proposed in Figure 3.

FTIR spectroscopy further confirmed the presence of the macromolecules within the hybrid compounds. The main band assignations are gathered in Table 3.

**Table 3:** Attribution of the main vibration bands of pristine and hybrid LDH phases.

MgAl-NO_3_	MgAl-PAA_49_-CTPPA	MgAl-P(AA*_n_*-*stat*-BA*_n_*)-CTPPA	Attribution

3430	3346	3430	ν OH
	2936	2961, 2932, 2877	νCH_3_, νCH_2_, νCH
		1720	νC=O (ester)
1647	1647	1647	δH_2_O
	1547, 1390	1568, 1390	ν_as_, ν_s_ –COO–
		1456	δCH_2_, δCH_3_
1350	1350	1350	νNO_3_
		1278, 1159	νC–O (ester)
		1068	νC=S
783, 651, 558	783, 651, 558	783, 651, 558	ν_Μ–Ο_
453	453	453	δ_Ο–Μ–Ο_

**Figure 4:**
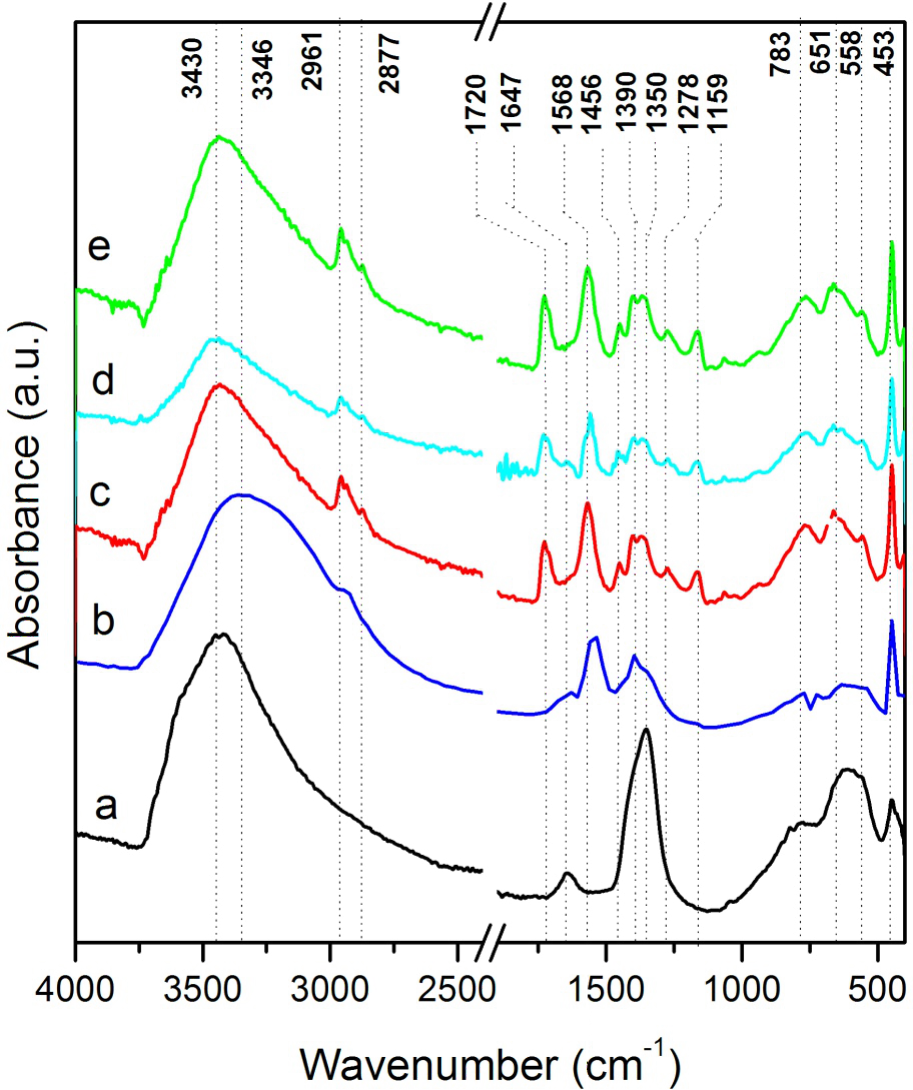
FTIR spectra of: a) pristine MgAl-NO_3_ and macroRAFT agent-intercalated LDHobtained by anion exchange in presence of b) PAA_49_-CTPPA, c) P(AA_8.5_-*stat*-BA_8.5_)-CTPPA, d) P(AA_14.5_-*stat*-BA_14.5_)-CTPPA and e) P(AA_19.5_-*stat*-BA_19.5_)-CTPPA.

As expected, all the FTIR spectra of the prepared compounds (Figure 4) exhibit the LDH characteristic bands [46] in particular those at 3700–3200 and 1647 cm^−1^ attributed to the stretching vibration of OH groups in the LDH layers and the stretching and bending mode of the water molecules, respectively.

In the low wavenumber region, the bands at 783, 651, 558 and 453 cm^−1^ can be attributed to the O–M–O and M–O vibration bands in the brucite-like layers. MacroRAFT (co)polymer intercalation was further confirmed by FTIR due to the intensity decrease of the nitrate vibration band at 1350 cm^−1^ (Figure 4), and the increasing intensities of new vibrations in both the 2961–2877 cm^−1^ region (νCH_3_, νCH_2_, νCH) and the 1750–1150 cm^−1^ region (νC–O, νC=O). For the PAA_49_-CTPPA-intercalated LDH, the shift of the OH vibration band at 3346 cm^−1^ in comparison with the precursor phase could traduce a modification of the hydrogen bond network in the interlayer domain due to macroRAFT polymer intercalation evidenced by the presence of the typical vibration of the carboxylate (ν_as_ and ν_s_). In the case of P(AA-*stat*-BA)-CTPPA copolymers, the nitrate vibration band is also replaced by the C–H, C=S, C=O, COO– stretching and bending modes of the macromolecules (Table 3).

**Figure 5:**
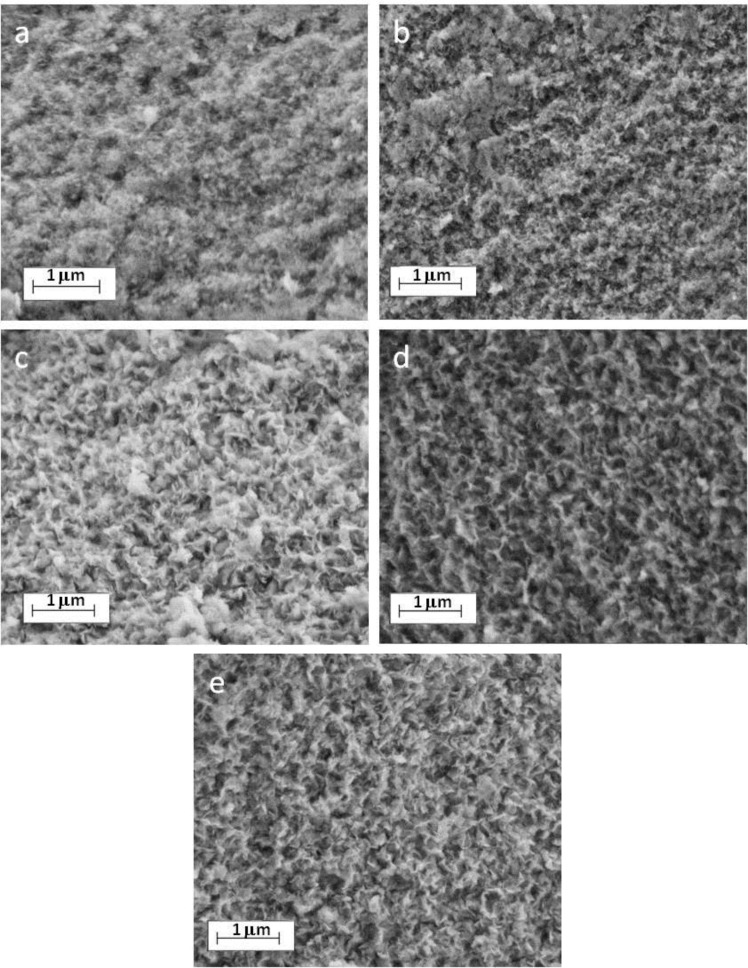
FESEM images of: a) pristine MgAl-NO_3_ LDH and macroRAFT agent-intercalated LDH obtained by anion exchange in presence of: b) PAA_49_-CTPPA, c) P(AA_8.5_-*stat*-BA_8.5_)-CTPPA, d) P(AA_14.5_-*stat*-BA_14.5_)-CTPPA and e) P(AA_19.5_-*stat*-BA_19.5_)-CTPPA.

As previously reported for macroRAFT copolymers adsorption onto LDHs [44], polymer intercalation systematically led to an inversion of the surface charges with ζ potential values close to −51 mV compared to the positive value of +43 mV measured for the MgAl-NO_3_ LDH precursor. This clearly evidences a modification of the particle surface through intercalation/adsorption phenomena. Interestingly, such a chemical and surface modification of LDH through anion exchange did not modify the particle shape and aggregation as evidenced by FESEM analysis (Figure 5). The different hybrid LDH compounds display similar platelet-like particle shapes aggregated in a house of cards morphology by edge-to-face associations.

**Figure 6:**
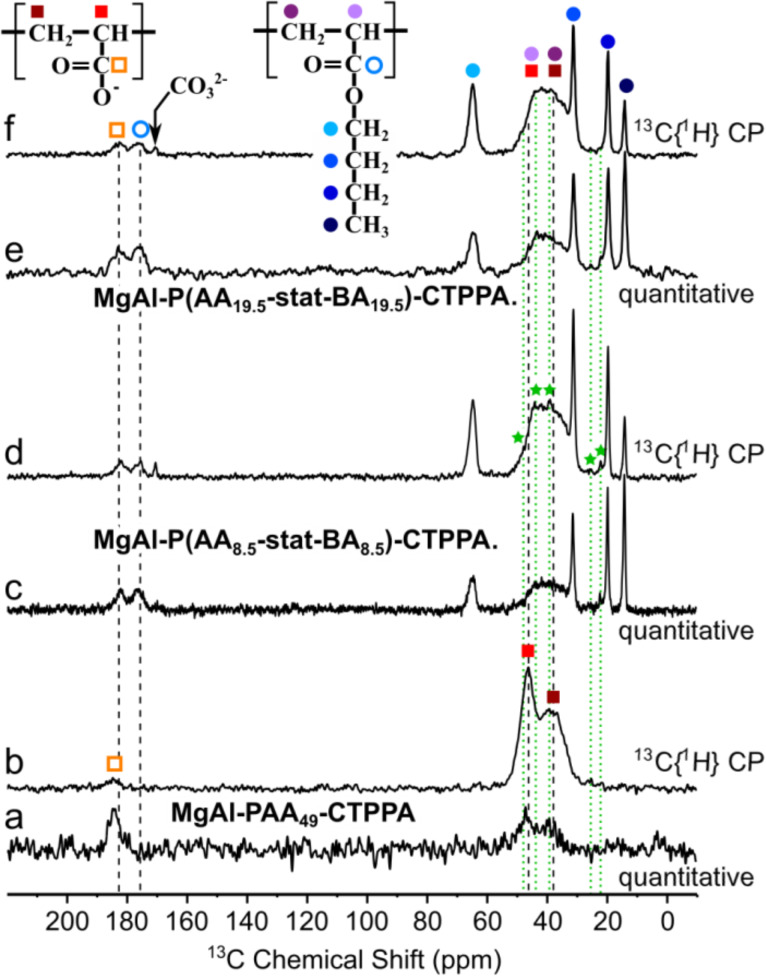
Solid-state ^13^C NMR spectra of (a,b) MgAl-PAA_49_-CTPPA; (c,d) MgAl-P(AA_8.5_-*stat*-BA_8.5_)-CTPPA and (e,f) MgAl-P(AA_19.5_-*stat*-BA_19.5_)-CTPPA. Spectra (a,c,e) are quantitative spin-echo experiments recorded in non-saturating quantitative conditions, whereas (b,d,f) correspond to ^13^C{^1^H} CP-MAS experiments recorded at short contact time (0.2 ms). Peak assignments are indicated by colored circles and squares for signals attributed to the AA and BA units, respectively. The green stars and dotted lines presumably correspond to C atoms within the terminal fragments of the copolymer.

To get further insight into the interaction between the macroRAFT (co)polymer and the LDH layer within the hybrid phases, solid state NMR experiments were also performed. One-dimensional (1D) ^13^C quantitative and ^13^C{^1^H} CP-MAS NMR spectra of the P(AA*_n_*-*stat*-BA*_n_*)-CTPPA materials are presented in Figure 6c–f along with similar spectra collected for PAA_49_-CTPPA (Figure 6a,b).

Comparisons of the spectra obtained for these two types of materials make it possible to distinguish and identify carbonyl signals from the AA and the BA units at 182 and 176 ppm, respectively. Higher shift of the AA carbonyl as compared to the BA carbonyl signal is consistent with stronger deshielding of the ^13^C nuclei due to the hydrogen bonding between C–O- and the LDH OH groups, which pulls electrons further away from the carbon, as compared to the butyl chain. In contrast with the PAA_49_-CTPPA system, where spectral contributions from backbone CH and CH_2_ carbon atoms are well distinguished (albeit overlapping), this backbone region is more severely crowded in the P(AA*_n_*-*stat-*BA*_n_*)-CTPPA spectra.

**Figure 7:**
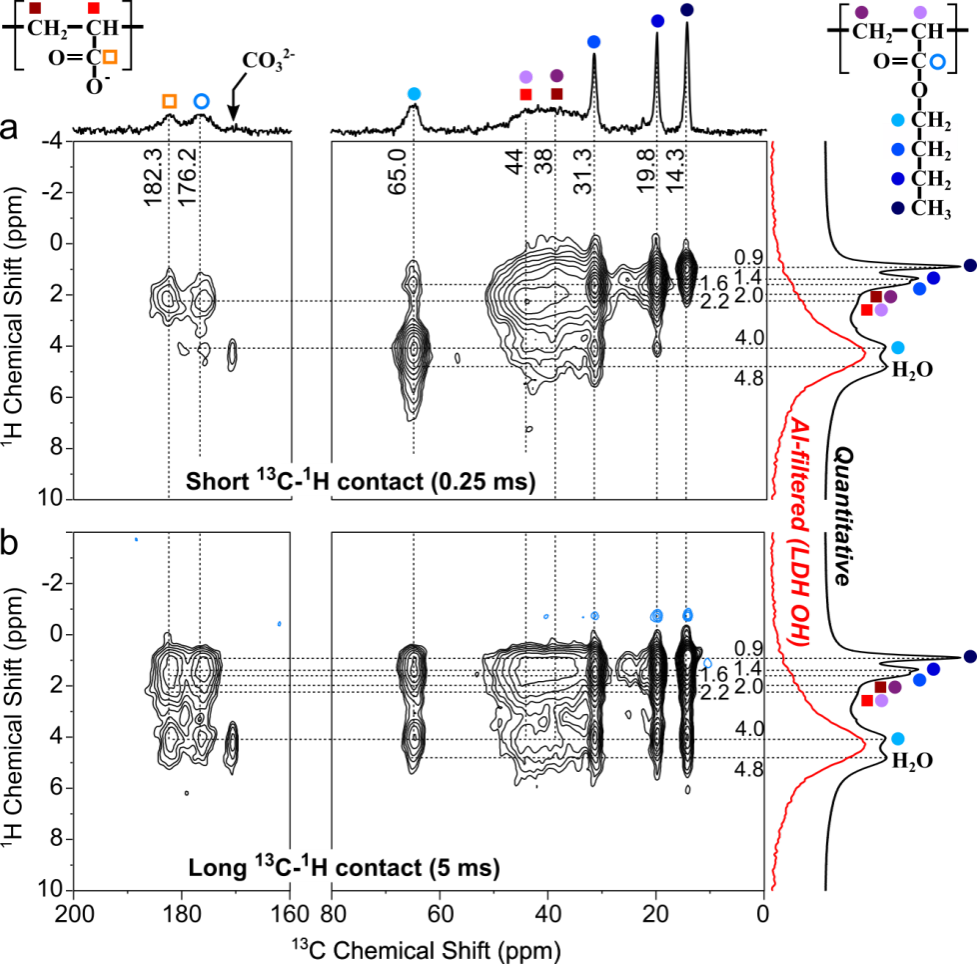
Solid-state ^13^C{^1^H} HETCOR NMR spectra of MgAl-P(AA_8.5_-*stat*-BA_8.5_)-CTPPA, and collected at: a) short CP contact time (0.25 ms) to emphasize short ^1^H–^13^C proximities (primarily C–H bonds) and b) at long CP contact time (5 ms) to allow magnetization transfer across longer ^1^H–^13^C distances. The 1D spectrum on the top is the quantitative ^13^C echo-MAS spectrum. 1D ^1^H spectra on the right were collected at fast (60 kHz) MAS to reduce broadening due to ^1^H–^1^H interactions: the black spectrum is a quantitative ^1^H echo-MAS and the red corresponds to a ^1^H{^27^Al} CP-MAS experiment selectively probing the LDH OH protons in close proximity to the framework Al sites.

Attempts to model this spectral region with 4 distinct (Gausso/Lorentzian) contributions, with or without constraints from the PAA_49_-CTPPA peak positions and/or widths, did not lead to sensible solutions. Best models were obtained with 3 lines, one at ca. 44 ppm attributed to CH groups (both from AA and BA units) and two additional contributions at ca. 39 and ca. 35 ppm representing CH_2_ groups, possibly one from AA and the other from BA units. Regardless of the model selected, the full width at half maximum (FWHM) of individual contributions was 5 ppm or more, which seems to indicate a large degree of chain conformational disorder. The comparison of echo-MAS quantitative (Figure 6a,c,e) and ^13^C{^1^H} CP spectra at short contact time (Figure 6b,d,f) did not show strong contrasts in the relative peak intensities, except for quaternary carbon atom (which were located further away from protons), which indicates homogeneous chain rigidity (or dynamics). There is no evidence, for example, of (co-)polymer domains being less immobilized by weaker interactions with the LDH surface or inter-chain interactions. Importantly, no significant difference can be observed between the AA and BA chain dynamics, except for the butyl group (full blue circles) whose faster re-orientational dynamics are illustrated by the very narrow peak widths of butyl CH_3_ (at 14 ppm) and middle-chain CH_2_ groups (at 22 and 20 ppm) in comparison with backbone CH_2_ groups (ca. 35–40 ppm). The butyl O-CH_2_ (at 65 ppm) shows only partial dynamic averaging due to its closer proximity to the more rigid backbone, indicating dynamics of the order of the peak width (ca. 10^3^ s^−1^). In addition, small signals detected at identical positions for different samples and marked with green stars and dotted lines in Figure 6 most likely correspond to some of the C atoms within end-chain fragments of the macroRAFT agents (see Figure 1). Two-dimensional (2D) ^13^C–^1^H correlation spectra exploiting ^13^C–^1^H proximities were collected to shed light on the ^1^H NMR peak assignments and provide further insights into the interactions between the (co)polymer guest and the LDH host. Figure 7 shows two ^13^C{^1^H} heteronuclear correlation (HETCOR) spectra based on the CP magnetization transfer between ^1^H and ^13^C nuclei, in which cross peaks indicate a spatial proximity between the ^1^H and ^13^C signals at the corresponding frequencies.

The two experiments probe different ranges of ^1^H–^13^C distances by means of different CP contact times: the short contact time (0.25 ms) used in Figure 7a reveals short ^1^H–^13^C distances: primarily (but not exclusively) C–H bonds, whereas the longer mixing time (5 ms) in Figure 7b is used to establish proximities between more distant ^1^H and ^13^C pairs (several Å to ca. 1 nm). The moderate spinning frequency (12 kHz) at which these spectra were collected should in principle yield very low resolution in the ^1^H dimension due to line broadening caused by dipole–dipole interactions that are too strong to be properly averaged by MAS. However, this is circumvented in the 2D HETCOR spectra by the application of homonuclear ^1^H decoupling during the ^1^H evolution period (see details in Experimental). For the same reason, the 2D peak positions in the ^1^H (vertical) dimension of the 2D spectra are compared to 1D ^1^H NMR spectra recorded at a faster spinning frequency (60 kHz) and shown on the right side. In both Figure 7a and 7b, the black spectrum corresponds to a quantitative ^1^H spin-echo experiment, whereas the red spectrum corresponds to a selective detection of ^1^H nuclei located in close proximity to the ^27^Al nuclei of the framework LDH host, i.e., primarily from the LDH OH groups, showing a broad peak at ca. 4.3

It was obtained by means of a ^1^H{^27^Al} CP-MAS experiment consisting in an excitation of ^27^Al nuclei followed by a CP transfer allowing through-space magnetization transfer from ^27^Al to ^1^H nuclei (via dipole–dipole couplings), for a time sufficiently short (0.5 ms) to selectively probe Al–O–H proximities within the LDH layers. 2D cross-peak positions along the ^13^C (horizontal) dimension are compared to the quantitative ^13^C NMR spectrum (same as in Figure 6c).

The cross peaks observed in the ^13^C{^1^H} HETCOR spectrum at short contact time (Figure 7a) and the associated increase in resolution facilitate the assignment of the otherwise poorly-resolved ^1^H (compared to ^13^C) NMR spectra. Assignment of ^1^H signals to the different protonated carbons within the copolymer are displayed in Figure 7. Even with this 2D spectrum, the ^1^H spectra are challenging to analyze due the overlap of the LDH OH signal at ca. 4.3 ppm with the butyl O–CH_2_^1^H signal at 4.0 ppm and the water signal at 4.8 ppm. There is also (as in ^13^C NMR) poor resolution within the backbone AA and BA CH_2_ and CH ^1^H signals (at ca. 2 ppm), which furthermore partially overlap with the butyl CH_2_ signals at 1.4 and 1.6 ppm. Nevertheless, the slightly tilted shape of the broad backbone correlation peak confirms that the ^13^C and ^1^H regions centered at ca. 44 and 2.2 ppm, respectively, are primarily due to the backbone CH of both AA and BA units. This ^1^H frequency indeed also correlates with the attached ^13^C carbonyl signals, consistent with this assignment. On the other hand, the contribution of ^13^C signals centered at ca. 38 ppm tends to correlate with a slightly shifted ^1^H signal at 2.0 ppm, which can thus be attributed to backbone CH_2_ groups of (undifferentiated) AA and BA units.

Despite these difficulties, the spectra can be analyzed in an attempt to gain insights into the (co)polymer interactions with the LDH surface. What we are seeking in particular are correlations between guest ^13^C nuclei and host hydroxyl protons. Carbonyl groups are expected to be most directly involved in such host–guest interactions. At short contact time (Figure 7a), the corresponding carbons (182 and 176 ppm for AA and BA units, respectively) are primarily found to be in close proximity to the backbone ^1^H protons (at 2.2 ppm), with also a small correlation between the BA carbonyl and the butyl O–CH_2_-protons. Only the carbonate signal at 171 ppm of the hydrotalcite(-like) impurity shows a clear correlation with the LDH OH groups at 4.2 ppm, in strong contrast with the very small amount of this impurity (less than 0.2% of the total C content). This indicates that host–guest interactions are considerably weaker in the (co)polymer-intercalated LDH material than in hydrotalcite. At longer contact times, correlations appear between the butyl ^1^H signals and both the AA and BA carbonyl ^13^C signals, which confirms the homogeneous (as opposed to segregated) repartition of AA and BA units along the polymer chains, such that most (if not all) AA units are located nearby BA units, permitting magnetization transfer from AA carbonyl ^13^C to nearby BA butyl protons. Among those, the butyl O–CH_2_ (4.0 ppm) ^1^H signal unfortunately overlaps with the LDH hydroxyl (4.3 ppm) and consequently prevents unambiguous observation of the spatial proximity between the guest carbonyl and the host hydroxyl groups. The same observations apply to the backbone ^13^CH and ^13^CH_2_ signals, which at long contact time also correlate with the butyl signal, similarly preventing the direct observation of a correlation with the LDH OH protons.

**Figure 8:**
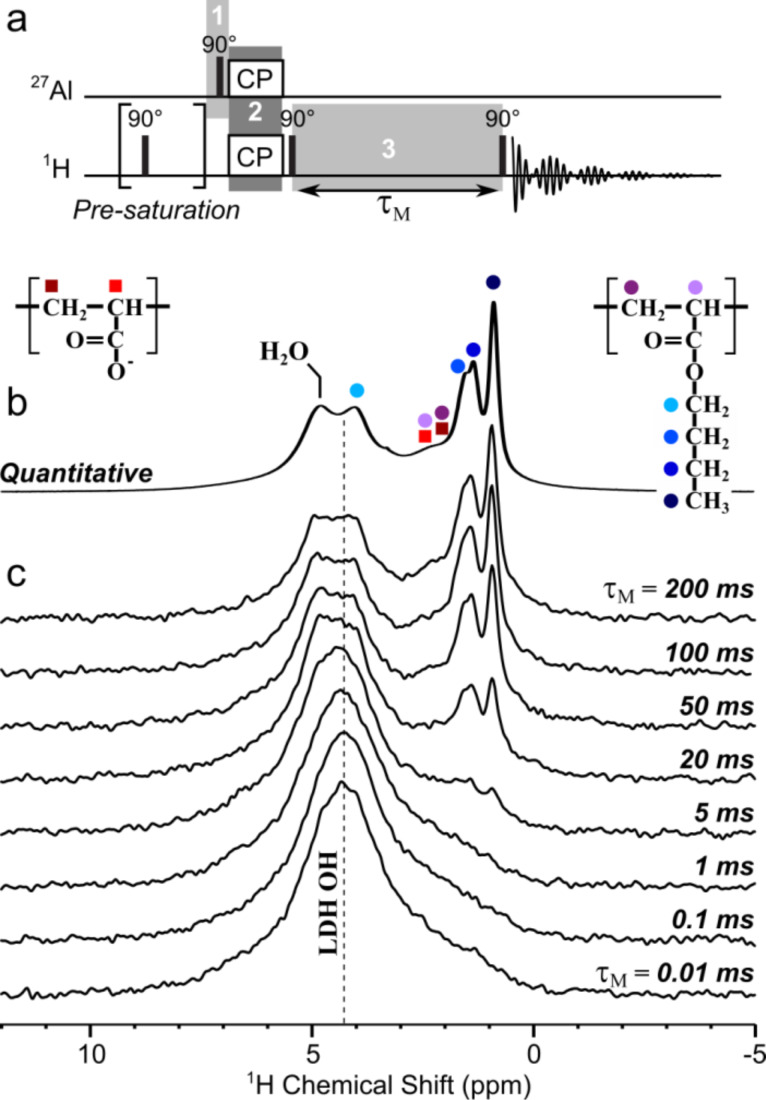
a) Radio-frequency-pulse NMR sequence used to probe LDH/copolymer interactions. Shaded regions labeled 1 to 3 correspond to the main three steps of the experiment, as described in the text. b) Quantitative solid-state ^1^H echo-MAS NMR spectrum of MgAl-P(AA_8.5_-stat-BA_8.5_)-CTPPA collected at 60 kHz MAS and 17.6 T. c) Series of ^1^H MAS NMR spectra collected (in the same conditions as in b)) with the NMR rf-pulse sequence shown in a) using spin-diffusion mixing times τ_M_ of 0.01 to 200 ms to allow progressive propagation of the magnetization from the selectively-excited LDH OH protons to other nearby protons.

An alternative way to more directly probe the interactions between the host LDH and the guest (co)polymer is to selectively create magnetization on the LDH Al site to first transfer it to nearby hydroxyl protons and then let it propagate to more remote protons (including those of the (co)polymer) using the strong ^1^H–^1^H dipolar couplings. This is done by means of a radio-frequency (rf)-pulse experiment shown in Figure 8a, which contains three main steps labeled 1, 2 and 3 therein. The ^27^Al magnetization is first created by exciting the LDH framework Al sites (by a pulse on the ^27^Al channel on Figure 8a). A short (0.5 ms) cross polarization (using rf irradiation on both ^1^H and ^27^Al channels simultaneously) then transfers this magnetization from ^27^Al to nearby protons, i.e., almost exclusively those of the LDH hydroxyl groups. In a last step, the obtained ^1^H magnetization is stored parallel to the external magnetic field axis for a time τ_M_ to allow this magnetization to diffuse among abundant ^1^H nuclei within both the LDH host and the (co)polymer guest. This process occurs through a magnetization exchange mechanism called spin-diffusion that results from ^1^H–^1^H dipolar interactions (hence the designation of τ_M_ as “spin-diffusion mixing time”). A final excitation pulse enables the detection of all the ^1^H nuclei to which magnetization has been transferred, depending on their proximity to the initial Al sites (and their connected OH) and the time during which the spin-diffusion processes. Similar approaches have been used to characterize, e.g., surfactants interacting with silica surfaces [47–48].

Figure 8c shows the results of a series of such an experiment conducted using different spin-diffusion mixing times for the P(AA_8.5_-*stat-*BA_8.5_)-CTPPA system, at fast MAS and high magnetic field to maximize spectral resolution. These can be compared with the quantitative ^1^H echo-MAS spectrum collected under the same conditions, shown in Figure 8b (same as shown in black on the right side of Figure 7). At shortest τ_M_ values (bottom), no significant diffusion of the ^1^H magnetization occurs, such that the spectrum selectively displays the LDH OH sites, which are located closest to the framework Al atoms (as in the ^27^Al{^1^H} CP-MAS spectrum shown in red on the right side of Figure 7).

As the mixing time increases, this initial magnetization propagates to increasingly long distances, which results in the progressive growth of all ^1^H NMR peaks associated with protons located close enough to the LDH layers, which includes the water signal at 4.8 ppm, the backbone CH and CH_2_ protons at 2.2 and 2.0 ppm (respectively), and the butyl CH_2_ (4.0, 1.6 and 1.4 ppm) and CH_3_ signals (0.9 ppm). Due to the low spectral resolution, it is difficult to tell whether some contributions are appearing before others. In fact the contributions to which slowest diffusion is expected are the mobile butyl protons (since mobility averages down the dipolar interactions), and yet these are the most clearly visible on the spectra because they are (for the same reason) the narrowest. Despite these limitations, it is clear that all ^1^H signals observed in the quantitative spectrum are indeed located nearby the LDH surface. The fact that we do not observe a particularly faster diffusion to the backbone CH signals (closest to the carbonyl groups) than to the other protons suggests that the interaction of the (co)polymer with the LDH surface is not particularly strong, consistent with the less-efficient CP transfer observed for the (co)polymer than for the residual carbonate (Figure 6 and Figure 7). Again, no clear distinction can be made between the AA and BA units, which is an important common feature of all NMR data reported here for the (co)polymer-intercalated LDHs (Figure 6, Figure 7 and Figure 8).

## Conclusion

In this study, hybrid materials were prepared by associating macromolecular macroRAFT anions with LDH inorganic layers through an anion exchange process from a nitrate LDH precursor phase. The full characterization of the hybrid materials evidenced a successful intercalation for both AA- and AA/BA-based macroRAFT polymers. Larger interlamellar distances were obtained in presence of BA units underlying the influence of the nature of the macroRAFT chain on the interlayer arrangement. Such difference indicates that the macroRAFT polymer involving only AA units was arranged as a monolayer in the interlamellar domain whereas the presence of BA units into the chain induced a bilayer arrangement whatever the chain length. Contrarily to what may be expected, based on NMR experiments, the negatively-charged AA units did not appear to interact sizeably more strongly with the LDH host than the BA units.

Our results demonstrate that hydrophilic macroRAFT polymers can be efficiently intercalated into MgAl-NO_3_ LDH forming a functional hybrid material which can be further involved in RAFT polymerization to promote grafting from polymerization or the formation of nanocomposite particles using an emulsion polymerization process [49].

## Experimental

Magnesium and aluminium nitrate salts, Mg(NO_3_)_2_·6H_2_O and Al(NO_3_)_3_·9H_2_O were of analytical grade (Acros Organics, Merck). Acrylic acid (AA, Aldrich, 99%), *n*-butyl acrylate (BA, 99%, stabilized, Acros Organics), 1,4-dioxane (Sigma-Aldrich, puriss. p.a., >99.5%), 1,3,5-trioxane (Sigma-Aldrich, >99%), the initiator 4,4-azobis(4-cyanopentanoic acid) (ACPA, Fluka, >98%), diethyl ether (Sigma-Aldrich, >99.5%) and NaOH (Acros Organics, Merck) were used without further purification. The RAFT agent: 4-cyano-4-thiothiopropylsulfanyl pentanoic acid (CTPPA) was synthesized following a protocol reported in the literature [50].

**Preparation of MgAl-NO_3_-LDH precursor.** LDH containing magnesium(II) and aluminium(III) metal cations and nitrate interlayer anions were prepared by flash coprecipitation followed by hydrothermal treatment [44,51–52]. Typically, a metallic nitrate solution (Mg/Al = 3; 0.3 M) was rapidly added to a NaOH (0.185 M) solution at 0 °C. The pH of the resulting suspension was adjusted to 9.5 and the solution was transferred to an autoclave and heated to 150 °C for 4 h. The resulting particles were collected by centrifugation and the resulting gel washed twice with deionized water. The nanoparticles were finally redispersed in deionized water and stored as a colloidal suspension (≈10 wt %) at room temperature.

**Synthesis of macroRAFT (co)polymers.** Four different hydrophilic polymers were synthesized by CTPPA-mediated RAFT polymerization of AA or statistical copolymerization of AA and BA (hereafter noted PAA*_n_*-CTPPA and P(AA*_n_*-*stat*-BA*_n_*)-CTPPA, respectively). Typically, AA, BA, CTPPA, ACPA and 1,3,5-trioxane, used as internal standard for NMR analysis, were dissolved in a round bottom flask, in 1,4-dioxane with a ratio of [monomer]/[RAFT]/[initiator] of 45:1:0.1 (mol/mol/mol) and a total monomer concentration of 3 and 6 M for AA homopolymerization and copolymerization with BA (AA/BA = 50:50 mol/mol), respectively. The solution was degassed with nitrogen for 30 minutes and placed in an oil bath at 80 °C. The reaction was conducted for 5 h and the obtained polymers were purified by precipitation in diethyl ether. Samples were taken during polymerization to determine conversion by ^1^H NMR spectroscopy as a function of time, and molar mass evolution with conversion by size exclusion chromatography (SEC). SEC measurements were carried out at 40 °C with a flow rate of 1 mL min^−1^ using toluene as a flow rate marker. Before analyses, carboxylic acid groups of the polymers were methylated in a THF/H_2_O (90:10 v/v%) mixture using tri(methylsilyl)diazomethane methylation agent to prevent interactions between acid groups and the stationary phase. Samples were filtered on a 0.45 μm pore size membrane and analyzed at 3 mg mL^−1^. Separation was carried out on three columns from Malvern Instruments (T6000 M General Mixed Org (300 × 8 mm)). The device (Viscotek TDA305) was equipped with a refractive index (RI) detector (λ = 670 nm). The number-average molar mass (*M*_n_) and dispersity (Đ = *M*_w_/*M*_n_), with *M*_w_: weight-average molar mass) were derived from the RI signal using a calibration curve based on polystyrene standards (from Polymer Laboratories) [53]. Table 1 presents the main characteristics of the well-defined polymers obtained with a narrow molar mass distributions (*Ð* = 1.15 ± 0.05) and further used in the intercalation process.

**MacroRAFT agent intercalation**. Standard anionic exchange was carried out at room temperature under nitrogen for 18 h. Typically, a gel mass corresponding to 0.1 g of dried MgAl-NO_3_ LDH was dispersed in 50 mL of water containing a twofold charge excess of macroRAFT agents per Al^3+^. Prior to be used, the aqueous macroRAFT agent solutions were neutralized by NaOH (0.5 M) addition at pH 8.0. The suspensions were stirred at a constant speed (800 rpm) and the products were recovered by centrifugation at 4500 rpm for 10 min followed by three washing cycles with deionized water and further dried at room temperature.

**Characterisations.** Powder X-ray diffraction patterns were recorded on a X’Pert Pro Philips diffractometer with a diffracted beam graphite monochromator and a Cu Kα radiation source in the 2θ range of 2–70°. The one-dimensional (1D) electron density distribution along the *c*-stacking axis ρ_(z)_ for MgAl-P(AA_8.5_-*stat-*BA_8.5_)-CTPPA was calculated from the intensity of the 00*l* diffraction lines according to the following equation:


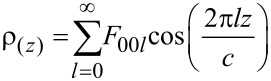


where *c* is the unit cell parameter, *z* is the fractional coordinate along the *c*-stacking axis, and *F*_00_*_l_* are the structure factors of the 00*l* diffractions [54]. Seven isolated 00*l* diffraction lines were used for calculating the 1D plot for X with a basal spacing of *d*_00_*_3_* ≈ 2.33 Å; the first peak corresponding to the 00*3* diffraction line was not considered being in the direct beam. First, the Le Bail-method consisting in the refinement of the total envelope of the XRD patterns was used to determine cell parameters assuming the *R*3*m* space group, typical for LDH materials, and to extract the intensities of diffraction peaks. The Thompson, Cox and Hastings (TCH) pseudo-Voigt function was chosen as profile function [55]. The background was refined by adjusting the height of preselected points for linear interpolation modeled and the spherical harmonics correction for an anisotropic peak broadening was applied. Afterwards, the hydroxide part of the structure was entered [56] and the *F*_00_*_l_* structure factors calculated; the signs of the structure factors were directly obtained from the scattering contributions of the Mg_3_Al(OH)_6_ hydroxide layers assuming a relatively small contribution of the intercalated molecules. The treatment of the XRD data was carried out using the Fullprof suite program [57]. ChemBio 3D Ultra suite program was used to draw the molecular structure of macroRAFT copolymers, running MM2 energy-minimization.

Attenuated total reflectance Fourier transform infrared (ATR-FTIR) spectra were measured in the range 400–4000 cm^−1^ on a FTIR Nicolet 5700 (Thermo Electron Corporation) spectrometer equipped with a Smart Orbit accessory. Field Emission SEM characteristics of the samples were imaged by a Zeiss supra 55 FEG-VP operating at 3 keV. Specimens were mounted on conductive carbon adhesive tabs and imaged after gold sputter coating to make them conductive. Zeta potentials of pristine LDH and macroRAFT-intercalated LDH were measured with a ZetaNano ZS (Malvern instruments) apparatus, using a laser Doppler electrophoresis. The measurements were performed in specific cells provided by Malvern Instruments Company.

Solid-state NMR spectra were collected on a Bruker 17.6 Tesla superconducting magnet operating at ^1^H, ^13^C, and ^27^Al Larmor frequencies of 750.10, 188.6, and 195.6 MHz. ^13^C experiments were performed at a magic-angle spinning (MAS) frequency of 12 kHz with a Bruker double-resonance 4 mm probehead. Cross-polarization (CP) contact times were set to 0.25 ms to favor short C–H distances, with 1024 scans for signal accumulation. Quantitative ^13^C experiments were collected with Hahn echo experiments at a total echo length of two rotor periods (167 μs), with a 50 s recycling delay to ensure non-saturating conditions. Signal accumulation was performed with 128 scans for the copolymer-intercalated LDH, and 32 scans for the PAA-intercalated material. Two-dimensional (2D) ^13^C{^1^H} heteronuclear correlation experiments used frequency-switched Lee–Goldburg (FSLG) homonuclear decoupling [58] at a nutation frequency of 75 kHz during the (indirect) ^1^H evolution period to reduce ^1^H–^1^H dipolar couplings and increase resolution. The associated frequency scaling factor (0.535) was measured and corrected for, based on a ^1^H–^1^H correlation experiment (spin-diffusion with null mixing time) conducted under identical homonuclear decoupling conditions. Contact times of 0.2 and 5 ms were used to observe contrast between short and long C–H distances, with (respectively) 512 and 480 scans for signal accumulation, 160 increments for the indirect dimension, and 1.2 s recycling delay (total acquisition time: 30 and 26 h, respectively).

Solid-state ^1^H NMR experiments were recorded with a Bruker 1.3 mm double-resonance probehead at the MAS frequency of 60 kHz. Quantitative echo experiments used a recycling delay of 5 s, ensuring non-saturating conditions, and a total echo length of two rotor periods (33 μs). ^1^H{^27^Al} CP-MAS experiments were performed with a short contact time of 0.5 ms (at constant amplitude on both channels) to ensure selective observation of the LDH hydroxyl protons (see [59] for further details on the application of this technique to MgAl LDHs). The signal was accumulated over 4096 transients with a recycle delay of 0.5 s. A 10-pulse pre-saturation on the ^1^H channel is used just before the initial ^27^Al excitation (90° pulse of 1.75 μs) to eliminate residual ^1^H signal, the absence of which was checked in an identical experiment in which the ^27^Al rf excitation pulse power was turned off. The same CP conditions were used for the ^1^H{^27^Al} CP-^1^H–^1^H spin-diffusion experiments, with the addition of two 90° pulses to first bring back the magnetization along the *z*-axis to allow spin-diffusion during mixing times τ_M_ of 0.01 to 200 ms, and to then send the resulting magnetization back to the transverse plane for detection. All experiments in the series used 4096 scans for signal accumulation with a recycle delay of 0.5 s. All ^13^C and ^1^H chemical shifts are referenced to (pure) tetramethylsilane.

## Supporting Information

Full PXRD patterns fitting using Le Bail method.

File 1 Additional PXRD analysis.
