# Supplementary material for: Intercalation and structural aspects of macroRAFT agents into MgAl layered double hydroxides
Source: Beilstein J Nanotechnol. 2016 Dec 15;7:2000–12. doi: 10.3762/bjnano.7.191 (PMC5238628; doi:10.3762/bjnano.7.191)
Supplement: File 1 — Additional PXRD analysis. [file Beilstein_J_Nanotechnol-07-2000-s001.pdf]

Supporting Information

for

Intercalation and structural aspects of macroRAFT agents into MgAl layered double hydroxides

Dessislava Kostadinova[1,2], Ana Cenacchi Pereira[3], Muriel Lansalot[3], Franck D'Agosto[3], Elodie Bourgeat-Lami[3], Fabrice Leroux[1,2], Christine Taviot-Guého[1,2], Sylvian Cadars[4] and Vanessa Prevot[1,2*]

Address: [1]Université Clermont Auvergne, Université Blaise Pascal, Institut de Chimie de Clermont-Ferrand, BP 10448, F-63000 Clermont-Ferrand, France, [2]CNRS, UMR 6296, ICCF, F-63171 Aubière, France, [3]Univ Lyon, Université Claude Bernard Lyon 1, CPE Lyon, CNRS 5265, Chemistry, Catalysis, Polymers and Processes (C2P2), 43 Bvd du 11 Novembre 1918, 69616 Villeurbanne, France and [4]Institut des Matériaux Jean Rouxel (IMN) - UMR6502, 2 rue de la Houssinière, BP32229 44322 Nantes cedex 3, France

Email: Vanessa Prevot - vanessa.prevot@univ-bpclermont.fr

* Corresponding author

Additional PXRD analysis

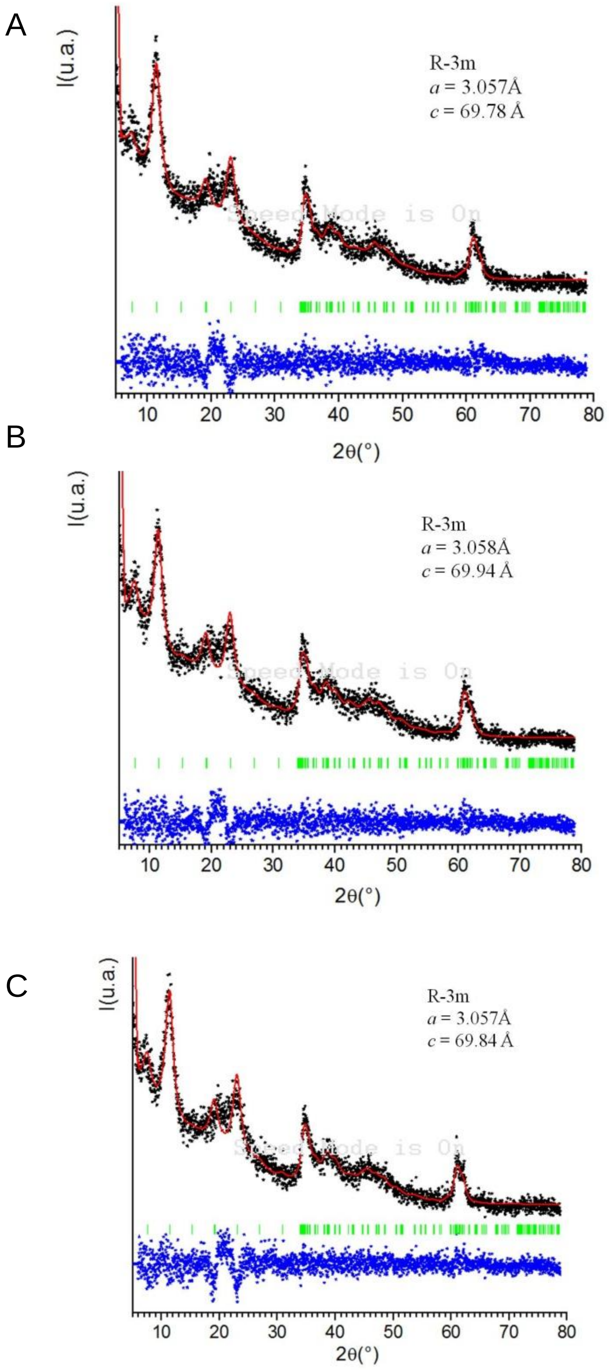

Figure S1: Results of the full pattern fitting of the X-ray diffraction pattern of macroRAFT intercalates using Le Bail method: (a) P($AA_{8.5}$-*stat*-$BA_{8.5}$)-CTPPA, (b) P($AA_{14.5}$-*stat*-$BA_{14.5}$)-CTPPA, (c) P($AA_{19.5}$-*stat*-$BA_{19.5}$)-CTPPA. The upper continuous red line is the calculated diffraction profile, the lower continuous blue line the difference between the experimental (black dots) and the calculated profiles. The vertical bars, at the bottoms, indicate the Bragg reflection positions expected in *R*3*m* space group.
